# Psoas muscle gauge and adverse clinical outcomes in patients on hemodialysis

**DOI:** 10.1007/s40620-024-02191-4

**Published:** 2025-01-28

**Authors:** Takahiro Yajima, Maiko Arao

**Affiliations:** Department of Nephrology, Matsunami General Hospital, Gifu, Japan

**Keywords:** Psoas muscle gauge, Hemodialysis, Mortality, New cardiovascular events

## Abstract

**Background:**

The relationship between the psoas muscle gauge (PMG), a combined sarcopenia indicator obtained from psoas muscle index (PMI) and psoas muscle density (PMD), and adverse clinical outcomes in patients on hemodialysis remains unclear. We examined whether psoas muscle gauge could predict all-cause mortality and new cardiovascular events more accurately than psoas muscle index in these patients.

**Methods:**

We retrospectively included 217 hemodialysis patients who underwent abdominal computed tomography. We calculated the psoas muscle gauge (arbitrary unit [AU]) at the fourth lumbar vertebra level as follows: PMI (cm^2^/m^2^) × PMD (Hounsfield units). We categorized the patients into higher and lower psoas muscle gauge groups based on sex-specific cutoffs obtained from the young Asian population. The outcomes were death and new cardiovascular events.

**Results:**

The psoas muscle gauge cutoffs were set at 231.1 and 328.8 AU in women and men, respectively. Eighty-five deaths and 95 new cardiovascular events occurred during the follow-up period of 4.4 (2.4–7.3) years. The 5-year survival rates were 59.2% and 94.9% in the lower and higher psoas muscle gauge groups, respectively (*p* < 0.0001). Moreover, after adjusting for sex and age, history of cardiovascular disease, C-reactive protein, modified creatinine index, and geriatric nutritional risk index, lower psoas muscle gauge was independently associated with increased all-cause death and new cardiovascular events (adjusted hazard ratio (aHR) 7.65; 95% confidence interval (CI) 2.37–24.66 and aHR 2.98; 95% CI 1.54–5.75, respectively). The concordance index (C-index) for predicting all-cause mortality and new cardiovascular events significantly improved when either psoas muscle index or psoas muscle gauge were added to the baseline risk model. Additionally, the C-index of the psoas muscle gauge-added model was significantly higher than that of the psoas muscle index-added model (0.815 vs. 0.784, *p* = 0.026) only when predicting all-cause mortality.

**Conclusions:**

Psoas muscle gauge accurately predicted the risk of all-cause mortality and new cardiovascular events in patients undergoing hemodialysis. For predicting all-cause mortality, psoas muscle gauge may be recommended compared to psoas muscle index.

**Graphical abstract:**

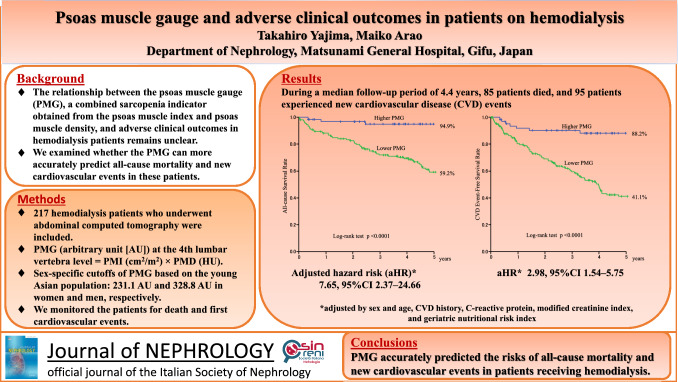

**Supplementary Information:**

The online version contains supplementary material available at 10.1007/s40620-024-02191-4.

## Introduction

Sarcopenia is diagnosed using a combination of low muscle quality and quantity and is highly prevalent in patients on dialysis (being found in 26.2–28.5% of the patients) [1–3]. Several recent systematic reviews and meta-analyses have revealed that sarcopenia, low muscle quantity, and low muscle quality are significantly associated with an increased risk of mortality in patients undergoing dialysis [3, 4]. For evaluating muscle quantity, bioelectrical impedance analysis or dual-energy X-ray absorptiometry is usually used, while handgrip strength (muscle strength) and/or gait speed (physical performance) are usually measured for evaluating physical function in clinical practice [5, 6]. Computed tomography (CT) is also recommended to assess sarcopenia in patients undergoing hemodialysis because it is less affected by the patients’ fluid volume status [7]. Height-adjusted psoas muscle thickness or abdominal skeletal muscle area is measured to evaluate muscle quantity [8, 9]. Skeletal muscle density, an average CT value in Hounsfield units (HU) of the muscle mass area, may be measured to evaluate muscle quality [8, 10]. We recently reported a close link between abdominal CT-based sarcopena indicators and mortality risk and/or occurrence of new cardiovascular events in patients on hemodialysis [11–14]. We have also recently documented that the psoas muscle index (PMI), an area of the psoas muscle divided by height-squared, and psoas muscle density (PMD), an average CT value of the psoas muscle area (PMA) measured at the L3 vertebra level, predicted the risk of all-cause death in dialysis patients [14]. According to the Asian Working Group for Sarcopenia (AWGS) 2019 [5], psoas muscle index and psoas muscle density cut-off values are favored to be determined by each mean value − 2 standard deviation (SD) of the young Asian population; therefore, the major limitation of our previous study was that only a reference range for psoas muscle index was reported at the L3 vertebra level in the young Asian population.

Recently, Lu et al. suggested the total psoas gauge (TPG), obtained by total psoas area (TPA) × HU average calculation (HUAC), as a predictor of prognosis post-radical gastrectomy in gastric cancer patients [15]. Tee et al. first proposed the term psoas muscle gauge (PMG), obtained by multiplying psoas muscle index by psoas muscle density [16]. Additionally, they provided sex-specific reference values of psoas muscle index and psoas muscle gauge but not psoas muscle density measured at the 4th lumbar (L4) vertebra level in the young Asian population. Conflicting results regarding the relationship between psoas muscle gauge and mortality have been reported in the fields of surgery and cancer [17–20]. Furthermore, the associations between psoas muscle gauge and adverse clinical outcomes in patients on hemodialysis remain unclear. We hypothesized that psoas muscle gauge may be clinically useful in predicting adverse clinical outcomes in hemodialysis patients.

Therefore, we examined the associations of psoas muscle gauge evaluated at the L4 vertebra level with all-cause mortality and cardiovascular events in patients on hemodialysis. We additionally investigated the predictive value of psoas muscle gauge compared to psoas muscle index when added to the baseline model in this population.

## Methods

### Study participants and ethical approval

Two-hundred forty-two patients receiving maintenance hemodialysis in Matsunami General Hospital located in Gifu Prefecture, Japan, who underwent plain abdominal CT for renal cancer screening from Jan. 2008 to Dec. 2019 were selected. The study protocol was conducted under the principles of the Declaration of Helsinki. It was approved by the Medical Ethics Committee of the Matsunami General Hospital (approval no: 543). The Medical Ethics Committee waived the need for informed consent because the present study was retrospectively designed using data from daily clinical practice at Matsunami General Hospital.

### Background data collection

Clinical information was obtained from medical charts, including sex; age; baseline kidney disease; vintage of hemodialysis; smoking habits; alcohol consumption; history of diabetes mellitus, hypertension, and cardiovascular disease (CVD); height and dry weight. Diabetes mellitus was defined by treatment with glucose-lowering agents and/or diabetic retinopathy, while hypertension was defined by blood pressure before hemodialysis sessions (diastolic blood pressure ≥ 90 mmHg and/or systolic blood pressure ≥ 140 mmHg) [21] and/or treatment with antihypertensive drugs. CVD was defined as having a history or the presence of peripheral artery disease, heart failure, angina pectoris, myocardial infarction, and brain infarction or hemorrhage. Blood samples were collected pre- and post-hemodialysis sessions at the beginning of the week. Laboratory data, including blood urea nitrogen, serum creatinine, albumin, hemoglobin, total cholesterol, and C-reactive protein (CRP), from the same month when CT was performed, were considered for analysis.

### Measurement of the psoas muscle area, psoas muscle index, psoas muscle density, and psoas muscle gauge at the L4 vertebra level

The free ImageJ ver. 1.53 (NIH, Bethesda, MD, USA; http://imagej.nih.gov/ij/) was used to analyze the imaging data [9]. A cross-sectional, non-enhanced CT image was selected at the level of the L4 vertebrae. The ‘Polygon selection tool’ was used to trace the border of the right and left psoas muscles, and the selected area and average CT value were automatically obtained. The CT value of the muscle threshold was set between -29 and 150 HU; the psoas muscle area was defined as the sum of the bilateral psoas muscle areas as follows: PMA = RtPMA (right PMA) + LtPMA (left PMA). The psoas muscle index was calculated as PMI (cm^2^/m^2^) = PMA (cm^2^)/height squared (m^2^); the psoas muscle density was calculated as PMD (HU) = [RtPMD (average CT value of the right psoas muscle) × RtPMA + LtPMD (average CT value of the left psoas muscle) × LtPMA]/PMA. Furthermore, the psoas muscle gauge (arbitrary units, AU) was defined as PMI (cm^2^/m^2^) × PMD (HU).

### Study design and endpoint

We categorized patients into the higher and lower psoas muscle gauge groups with three sex-specific cutoff values of psoas muscle gauge as follows: 1, cutoff values based on the values of the healthy young Asian population for each sex (mean-2SD); 2, cutoff values based on the median values in each sex; and 3, cutoff values based on the values obtained using the ROC curve analysis for maximally predicting all-cause death in each sex. Similarly, the patients were classified into higher and lower psoas muscle index groups.

We followed-up these patients until December 2021 and monitored mortality and cardiovascular events. We set the study endpoint as the time to death and the first cardiovascular event. In the present study, cardiovascular events were defined as cardiovascular diseases requiring hospitalization for treatment, such as arrhythmia, heart failure, angina, myocardial infarction, sudden death, stroke, and peripheral artery disease.

### Statistical analyses

Normally distributed data were described as mean ± SD, and non-normally distributed data were described as median and interquartile range. Malnutrition was defined as geriatric nutritional risk index < 91.2 [22]. We used Mann–Whitney *U* or *t* test and chi-squared tests to analyze continuous and categorical variables. We performed univariate regression analysis to evaluate the relationship between psoas muscle gauge and baseline variables. Thereafter, we performed multivariate regression analysis with a model that included significant variables in the univariate analysis. We performed ROC analysis with the Youden index and obtained the sex-specific cutoff values of psoas muscle gauge for maximally predicting all-cause mortality.

We used the Kaplan–Meier method for estimating all-cause and new cardiovascular event-free survival rates in the lower and higher psoas muscle gauge groups divided by three different cutoff values and analyzed the differences with the log-rank test. We used univariate Cox regression analysis to estimate the risks of all-cause mortality and new cardiovascular events (hazard ratios (HRs) and 95% confidence intervals (CIs)). We used a multivariate Cox analysis with the model including sex and significant variables in the univariate Cox analysis. We evaluated whether the predictive value for estimating mortality and new cardiovascular events could improve when psoas muscle index and psoas muscle gauge (both binary with the cut-offs of the Asian population) were added to the baseline risk model, including sex and significant variables in the univariate Cox regression analysis (*p* < 0.05). Additionally, we added subgroup analyses based on gender (male and female) and age (< 65 and ≥ 65 years). The C-index, which is the area under the ROC curve (AUC), was calculated and compared using the DeLong test [23] between the baseline risk model and the models enriched with either psoas muscle index or psoas muscle gauge (both binary). In this study, the analysis was performed only with the available data; therefore, imputation of missing data was not performed. Statistical significance was set at *p* < 0.05. We used R ver. 4.04 and SPSS Statistics, ver. 28 (IBM Corp., Armonk, N.Y., USA) for statistical analysis.

## Results

### Patients’ characteristics

Twenty-five patients had a history of malignancy; therefore, we enrolled 217 patients (age: 62.7 ± 13.7 years; male: 66.4%) in this retrospective study (Table 1). The median duration of hemodialysis was 22.0 (10.8–60.0) months, and 63.7% of patients had a history of CVD. CRP, modified creatinine index, and geriatric nutritional risk index were 0.16 (0.07–0.54) mg/dL, 20.6 ± 3.1 mg/kg/day, and 93.5 ± 8.4, respectively. When malnutrition was defined by the geriatric nutritional risk index, 31.3% of the patients were categorized as having malnutrition. Psoas muscle area, psoas muscle index, psoas muscle density, and psoas muscle gauge were 11.7 ± 6.0 cm^2^, 4.4 ± 2.1 cm^2^/m^2^, 40.3 ± 9.4 HU, and 219.0 (149.3–311.6) AU, respectively.

**Table 1:** Participants’ baseline information categorized into higher and lower psoas muscle gauge groups with cut-off values of the young Asian population

	All patients (*n* = 217)	Lower psoas muscle gauge group^a^ (*n* = 153)	Higher psoas muscle gauge group^b^ (*n* = 64)	*p* value
Age (years)	62.7 ± 13.7	66.2 ± 11.6	54.4 ± 14.9	< 0.0001
Sex, Male (%)	66.4	62.7	75.0	0.077
Baseline kidney disease				
Diabetic nephropathy (%)	42.9	37.3	56.2	0.071
Chronic glomerulonephritis (%)	30.0	32.0	25.0	
Nephrosclerosis (%)	19.3	21.6	14.1	
Others (%)	7.8	9.2	4.7	
Hemodialysis vintage (months)	22.0 (10.8–60.0)	27.3 (10.8–61.4)	18.3 (10.8–47.5)	0.18
Hypertension (%)	94.5	93.5	96.9	0.29
Diabetes (%)	46.1	41.8	56.3	0.052
Cardiovascular disease history (%)	65.0	69.3	54.7	0.042
Body mass index (kg/m^2^)	22.2 ± 4.1	21.3 ± 3.7	24.3 ± 4.3	< 0.0001
Blood urea nitrogen (mg/dL)	57.6 ± 14.6	56.1 ± 15.6	61.1 ± 11.5	0.022
Creatinine (mg/dL)	9.4 ± 3.1	8.9 ± 2.8	10.5 ± 3.6	0.0004
Albumin (g/dL)	3.7 ± 0.5	3.6 ± 0.5	3.9 ± 0.3	0.0001
Hemoglobin (g/dL)	10.5 ± 1.4	10.4 ± 1.6	10.9 ± 0.9	0.031
Total cholesterol (mg/dL)	150 ± 34	149 ± 33	152 ± 35	0.63
C-reactive protein (mg/dL)	0.16 (0.07–0.54)	0.20 (0.09–0.64)	0.11 (0.04–0.21)	< 0.0001
Modified creatinine index (mg/kg/day)	20.6 ± 3.1	19.9 ± 2.7	22.1 ± 3.5	< 0.0001
Geriatric nutritional risk index	93.5 ± 8.4	91.7 ± 9.0	97.8 ± 4.9	< 0.0001
Psoas muscle area (cm^2^)	11.7 ± 6.0	11.6 ± 4.2	20.5 ± 5.3	< 0.0001
Psoas muscle index (cm^2^/m^2^)	4.4 ± 2.1	4.5 ± 1.4	7.6 ± 1.6	< 0.0001
Psoas muscle density (Hounsfield units)	40.3 ± 9.4	38.1 ± 8.9	49.7 ± 3.8	< 0.0001
Psoas muscle gauge (Arbitrary units)	219.0 (149.3–311.6)	171.6 (123.8–229.1)	372.8 (333.9–424.1)	< 0.0001

^a^Lower psoas muscle gauge group: women with psoas muscle gauge of < 231 AU and men with psoas muscle gauge of < 328 AU

^b^Higher psoas muscle gauge group: women with psoas muscle gauge of ≥ 231 AU and men with psoas muscle gauge of ≥ 328 AU

### Associations of psoas muscle gauge with baseline variables

Psoas muscle gauge was significantly correlated with male sex, age, CVD history, modified creatinine index, geriatric nutritional risk index, and log-transformed CRP. Moreover, psoas muscle gauge was independently associated with male sex (*β* = 0.361: 95% CI 0.243–0.477, *p* < 0.0001), age (*β* = − 0.325: 95% CI − 0.473 to − 0.176, *p* < 0.0001), and geriatric nutritional risk index (*β* = 0.216: 95% CI 0.088–0.343, *p* = 0.0010) (Supplementary Table 1).

### Three different cut-off values of psoas muscle index and psoas muscle gauge

Regarding psoas muscle index, the psoas muscle index of men and women in the healthy young Asian population was 11.26 ± 2.26 and 7.70 ± 1.39 cm^2^/m^2^, respectively. We defined the sex-specific cutoffs of the Asian population as “mean-2SD”; therefore, these were 6.75 in men and 4.91 cm^2^/m^2^ in women. In this study, the psoas muscle index was 5.86 (4.70–7.50) and 4.11 (2.82–5.01) cm^2^/m^2^ in men and women, respectively; therefore, we set the sex-specific median cutoffs of psoas muscle index as 5.86 in men and 4.11 cm^2^/m^2^ in women. Finally, we conducted a ROC curve analysis to obtain sex-specific cutoff values for maximally predicting all-cause mortality. The cutoffs were 4.98 (AUC = 0.801, sensitivity = 0.864, and specificity = 0.589, *p* < 0.0001) in men and 3.39 (AUC = 0.727, sensitivity = 0.773, and specificity = 0.655, *p* = 0.0007) cm^2^/m^2^ in women. Therefore, we set these values as the sex-specific ROC-derived cutoffs for maximally predicting all-cause mortality.

### Associations of the psoas muscle index and psoas muscle gauge with all-cause mortality and new cardiovascular events

Eighty-five patients died, and 95 developed new cardiovascular events during a median follow-up period of 4.4 (2.4–7.3) years. The causes of death were CVD (*N* = 47, 55.3%), infection (*N* = 19, 22.4%), malignancy (*N* = 6, 7.0%), and others (*N* = 13, 15.3%). The new cardiovascular events were arrhythmia (*N* = 6, 6.3%), heart failure (*N* = 14, 14.7%), angina (*N* = 29, 30.5%), myocardial infarction (*N* = 6, 6.3%), sudden death (*N* = 3, 3.2%), stroke (*N* = 25, 26.3%), and peripheral artery disease (N = 12, 12.6%).

**Fig. 1:**
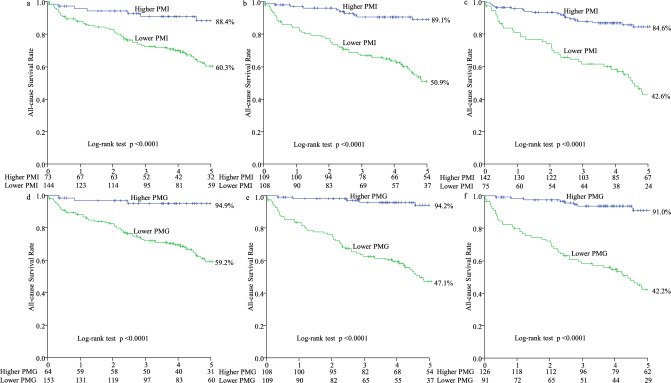
Kaplan–Meier survival curves for estimating all-cause mortality. All-cause mortality rates for comparing the lower and higher psoas muscle index (PMI) groups based on the (a) sex-specific cut-off values of the healthy young Asian population^1^, (b) sex-specific median values^2^, and (c) sex-specific cut-off values obtained using the receiver operating characteristic (ROC) curve analysis^3^. All-cause mortality rates for comparing the lower and higher psoas muscle gauge (PMG) groups based on the (d) sex-specific cut-off values of the healthy young Asian population^4^, (e) sex-specific median values^5^, and (f) sex-specific cut-off values obtained using the ROC curve analysis^6^. ^1^Cut-off values based on the PMI values of the healthy young Asian population: women, PMI of < 4.91 cm^2^/m^2^; men, PMI of < 6.75 cm^2^/m^2^. ^2^Cut-off values based on the median values of PMI in this cohort: women, PMI of < 4.11 cm^2^/m^2^; men, PMI of < 5.86 cm^2^/m^2^. ^3^Cut-off values based on the values obtained using the ROC curve analysis for maximally predicting all-cause mortality: women, PMI of < 3.39 cm^2^/m^2^; men, PMI of < 4.98 cm^2^/m^2^. ^4^Cut-off values based on the values of the healthy young Asian population: women, PMG of < 231.1 arbitrary units (AU); men, PMG of < 328.8 AU. ^5^Cut-off values based on the median values of PMG in this cohort: women, PMG of < 164.0 AU; men, PMG of < 252.1 AU. ^6^Cut-off values based on the values obtained using the ROC curve analysis for maximally predicting all-cause mortality: women, PMG of < 107.7 AU; men, PMG of < 246.3 AU

When the sex-specific cutoffs of psoas muscle index and psoas muscle gauge were set based on the reference values of the young Asian population, the 5-year survival rates were 88.4% and 60.3% in the higher and lower psoas muscle index groups, respectively, and 94.9% and 59.2% in the higher and lower psoas muscle gauge groups, respectively (both *p* < 0.0001) (Fig. 1a, d). The 5-year new cardiovascular event-free survival rates were 80.3% and 43.2% in the higher and lower psoas muscle index groups, respectively, and 88.2% and 41.1% in the higher and lower psoas muscle gauge groups, respectively (both *p* < 0.0001) (Supplementary Fig. 1a and 1d). Similar results were obtained when the cutoffs of psoas muscle index and psoas muscle gauge were set at the median values and the ROC-derived value for maximally predicting all-cause mortality (Fig. 1b, c, e, f and supplementary Fig. 1b, c, e, f, respectively).

After adjusting for sex and age, CVD history, CRP, modified creatinine index, and geriatric nutritional risk index, lower psoas muscle index (Asian population-specific cut-off value) was independently associated with an increased risk of all-cause mortality (adjusted HR (aHR) = 1.99, 95% CI 1.03–3.85, *p* = 0.040) and new cardiovascular events (aHR = 1.93, 95% CI 1.11–3.35, *p* = 0.019). Similarly, lower psoas muscle gauge (Asian population-specific cut-off value) was independently associated with an increased risk of all-cause mortality (aHR = 7.65, 95% CI 2.37–24.66, *p* = 0.0007) and new cardiovascular events (aHR = 2.98, 95% CI 1.54–5.75, *p* = 0.0012). The results were similar when patients were categorized into the higher and lower psoas muscle index and higher and lower psoas muscle gauge groups using the sex-specific cutoff values with medians and those derived from the ROC analysis, respectively (Table 2).

**Table 2:** Cox proportional hazards analysis of psoas muscle index and psoas muscle gauge for all-cause mortality and new cardiovascular events

Variables	Univariate analysis		Multivariate analysis^a^	
	HR (95% CI)	*p* value	HR (95% CI)	*p* value
All-cause mortality				
Lower psoas muscle index (Asian)^b^	3.86 (2.05–7.28)	< 0.0001	1.99 (1.03–3.85)	0.040
Lower psoas muscle index (median)^c^	3.63 (2.24–5.89)	< 0.0001	1.77 (1.04–3.00)	0.033
Lower psoas muscle index (ROC-derived)^d^	4.07 (2.62–6.32)	< 0.0001	2.24 (1.35–3.71)	0.0017
Lower psoas muscle gauge (Asian)^e^	14.70 (4.64–46.61)	< 0.0001	7.65 (2.37–24.66)	0.0007
Lower psoas muscle gauge (median)^f^	7.89 (4.34–14.33)	< 0.0001	4.05 (2.13–7.69)	< 0.0001
Lower psoas muscle gauge (ROC-derived)^g^	8.08 (4.74–13.77)	< 0.0001	5.06 (2.76–9.27)	< 0.0001
New cardiovascular events				
Lower psoas muscle index (Asian)^b^	2.98 (1.76–5.05)	< 0.0001	1.93 (1.11–3.35)	0.019
Lower psoas muscle index (median)^c^	2.87 (1.86–4.41)	< 0.0001	1.79 (1.11–2.87)	0.016
Lower psoas muscle index (ROC-derived)^d^	2.55 (1.69–3.83)	< 0.0001	1.62 (1.02–2.57)	0.043
Lower psoas muscle gauge (Asian)^e^	4.73 (2.52–8.91)	< 0.0001	2.98 (1.54–5.75)	0.0012
Lower psoas muscle gauge (median)^f^	3.74 (2.39–5.85)	< 0.0001	2.41 (1.46–3.95)	0.0005
Lower psoas muscle gauge (ROC-derived)^g^	3.97 (2.59–6.09)	< 0.0001	2.78 (1.69–4.56)	< 0.0001

*HR* hazard ratio, *CI* confidence interval, *ROC* receiver operating characteristic

^a^Adjusted by sex and age, cardiovascular disease history, C-reactive protein, modified creatinine index, and geriatric nutritional risk index

^b^Cut-off values based on the psoas muscle index values of the healthy young Asian population: women, psoas muscle index of < 4.91 cm^2^/m^2^; men, psoas muscle index of < 6.75 cm^2^/m^2^

^c^Cut-off values based on the median values of psoas muscle index in this cohort: women, psoas muscle index of < 4.11 cm^2^/m^2^; men, psoas muscle index of < 5.86 cm^2^/m^2^

^d^Cut-off values based on the values obtained by ROC curve analysis for maximally predicting all-cause mortality: women, psoas muscle index of < 3.39 cm^2^/m^2^; men, psoas muscle index of < 4.98 cm^2^/m^2^

^e^Cut-off values based on the values of the healthy young Asian population: women, psoas muscle gauge of < 231.1 arbitrary units (AU); men, psoas muscle gauge of < 328.8 AU

^f^Cut-off values based on the median values of psoas muscle gauge in this cohort: women, psoas muscle gauge of < 164.0 AU; men, psoas muscle gauge of < 252.1 AU

^g^Cut-off values based on the values obtained by ROC curve analysis for maximally predicting all-cause mortality: women, psoas muscle gauge of < 107.7 AU; men, psoas muscle gauge of < 246.3 AU

### Associations of psoas muscle index and psoas muscle gauge with all-cause mortality and new cardiovascular events in subgroups by gender and age

Lower psoas muscle index tended to increase the risk of all-cause mortality and new cardiovascular events, when patients were divided into subgroups based on gender (male and female) and age (< 65 and ≥ 65 years), respectively (Supplemental Table 2). Similar results were obtained for psoas muscle gauge (Supplemental Table 3).

**Table 3:** Predictions based on psoas muscle index and psoas muscle gauge for all-cause mortality and new cardiovascular events

Variables	C-index	*p* value
All-cause mortality		
Baseline risk model^a^	0.746 (0.680–0.813)	
+ Psoas muscle index (binary, cut-offs with the Asian population)	0.784 (0.721–0.846)	0.042
+ Psoas muscle gauge (binary, cut-offs with the Asian population)	0.815 (0.759–0.871)*	0.0006
New cardiovascular events		
Baseline risk model^a^	0.743 (0.678–0.809)	
+ Psoas muscle index (binary, cut-offs with the Asian population)	0.783 (0.721–0.845)	0.035
+ Psoas muscle gauge (binary, cut-offs with the Asian population)	0.793 (0.733–0.854)**	0.020

^a^Baseline risk model included sex and age, cardiovascular disease history, C-reactive protein, modified creatinine index, geriatric nutritional risk index

^*^p value = 0.026; compared to baseline risk model + psoas muscle index

^**^p value = 0.34; compared to baseline risk model + psoas muscle index

### Model discrimination

The C-index significantly improved from 0.746 to 0.784 (*p* = 0.042) and 0.815 (*p* = 0.0006) when psoas muscle index (binary) and psoas muscle gauge (binary) were added to the baseline risk model, including sex, age, history of CVD, CRP, modified creatinine index, and geriatric nutritional risk index, respectively. As for new cardiovascular events, similar results were obtained (Table 3). Additionally, the C-index of the model that included psoas muscle gauge was significantly higher than that of the model that included psoas muscle index (*p* = 0.026) only when predicting all-cause mortality (Table 3).

## Discussion

Lower psoas muscle gauge was independently associated with all-cause mortality and new cardiovascular events in patients on hemodialysis. The predictive values for all-cause mortality and new cardiovascular events significantly improved after either psoas muscle gauge or psoas muscle index was added to the baseline risk model. Additionally, the predictive value of psoas muscle gauge was superior to that of psoas muscle index for all-cause mortality; therefore, we recommend evaluation of psoas muscle gauge rather than psoas muscle index alone in these patients.

We have recently reported that psoas muscle index was independently associated with skeletal muscle index estimated using bioimpedance analysis in patients on hemodialysis [9]. Cheema et al. reported that thigh myosteatosis, evaluated using CT scan, was associated with gait speed in these patients [24]. These findings suggest that the psoas muscle index and psoas muscle density may reflect muscle quantity and quality, respectively. Geriatric nutritional risk index, a combined marker of body mass index and serum albumin level, is a marker of malnutrition that is easily calculated, enables serial evaluation, and is a powerful indicator of mortality in these patients [22, 25–27]. Protein-energy wasting, a well-known phenotype of malnutrition defined as muscle and fat wasting, is correlated with various adverse clinical outcomes, including mortality [1, 28]. In this study, psoas muscle gauge was independently associated with geriatric nutritional risk index, therefore psoas muscle gauge may be a surrogate marker of protein-energy wasting.

Six previous studies evaluated the association between psoas muscle gauge and mortality. Lu et al. first suggested that the psoas muscle gauge measured at the L3 vertebra level predicted complications, prognosis, and liver recurrence detection post-radical gastrectomy in gastric cancer patients [15]. In their study, psoas muscle gauge alone, rather than psoas muscle index or psoas muscle density, predicted cancer-specific, recurrence-free, and overall survivals. Tee et al. first named psoas muscle gauge and provided the sex-specific reference values of psoas muscle index and psoas muscle gauge measured at the L4 vertebra level in a young Asian population [16]. They demonstrated that low psoas muscle gauge independently predicted postoperative complications and mortality risks in geriatric patients with abdominal emergencies. Wu et al. reported that psoas muscle index and psoas muscle gauge, rather than psoas muscle density, measured at the L3 vertebra level were independently correlated with surgery-related clinical outcomes and 30-day mortality in patients who underwent emergency laparotomy [17]. Thormann et al. demonstrated that the psoas muscle index, psoas muscle density, and psoas muscle gauge at the L3 vertebra level were not predictors of overall survival in patients undergoing interstitial brachytherapy for breast or colorectal cancer liver metastasis [18, 19]. In addition, Zhang et al. reported that psoas muscle gauge at the L3 vertebra level was not associated with 1-year cancer-related mortality [20]. This current study revealed that psoas muscle gauge measured at the L4 vertebra level was an independent predictor of all-cause mortality and new cardiovascular events in patients on hemodialysis. Additionally, subgroup analysis based on gender and age revealed that a lower psoas muscle gauge tended to increase the risk of these adverse clinical outcomes. Moreover, the predictive accuracy of psoas muscle gauge for all-cause mortality was higher than that of psoas muscle index in these patients. Several studies have reported that psoas muscle index is an independent predictor of mortality in these patients [29, 30]. We recently reported that the psoas muscle index and psoas muscle density measured at the L3 vertebra level can accurately predict all-cause mortality in these patients [14]. Therefore, this study similarly demonstrates the importance of combining psoas muscle index and psoas muscle density, rather than psoas muscle index alone, with a simpler new integrated sarcopenic indicator of psoas muscle gauge.

Setting cutoff values of psoas muscle gauge remains debatable. Two studies measured psoas muscle gauge at the L3 vertebra level and set its cut-off values at the lowest quartile; the cohort-specific cut-off values were 219.4 and 118.4 AU for men and women, respectively, in patients with gastric cancer [15] and 169.7 and 106.7 AU for men and women, respectively, in those who underwent emergency laparotomy [17]. Conversely, a study measured psoas muscle gauge at the L4 vertebra level and set its cut-off values at mean-2SD based on the reference value of the young Asian population: 328.8 and 231.1 AU for men and women, respectively [16]. The cut-off values based on the reference values of the Asian population are likely larger than those based on the cohort of patients with specific diseases. In this study, a similar trend was confirmed. However, similar results with a setting at each of the three sex-specific cutoff points may support the robustness of our study results.

This study has some limitations. First, it was retrospectively performed, therefore, a sample size calculation was not performed. Second, this single-center retrospective study enrolled a small number of Japanese patients on hemodialysis. Therefore, the findings cannot be generalized to all hemodialysis populations. Third, the sex-specific cutoffs of psoas muscle gauge were adopted from those of the young Asian population; therefore, further studies are needed in the Western population. Fourth, psoas muscle index, psoas muscle density, and psoas muscle gauge were measured at the L4 vertebra level. In the future, if the reference values of these sarcopenia indices measured at the L3 vertebra level, the most common measurement location [8], are obtained, re-evaluation may be required. Finally, psoas muscle gauge was measured only at the inclusion; therefore, changes in the psoas muscle gauge could not be considered. Further prospective multicenter, large-scale studies that include other ethnic groups are needed to validate our study results.

In conclusion, psoas muscle gauge was found to be an independent predictor of all-cause mortality and new cardiovascular events in patients undergoing hemodialysis. Moreover, the predictive power of psoas muscle gauge, especially for all-cause mortality, was significantly higher than that of psoas muscle index. Therefore, psoas muscle gauge assessment, rather than psoas muscle index alone, may be recommended as a predictor of mortality in this population.

## Supplementary Information

Below is the link to the electronic supplementary material.

Supplementary file1 (DOCX 24 KB)

Supplementary file2 (PPTX 87 KB)

## Data Availability

The datasets generated and/or analyzed during the present study are available from the corresponding author upon reasonable request.
