# Supplementary material for: Psoas muscle gauge and adverse clinical outcomes in patients on hemodialysis
Source: J Nephrol. 2025 Jan 28;38(2):655–64. doi: 10.1007/s40620-024-02191-4 (PMC11961518; doi:10.1007/s40620-024-02191-4)
Supplement: Supplementary file 1 — Supplementary file1 (DOCX 24 KB) [file 40620_2024_2191_MOESM1_ESM.docx]

**Supplementary materials**

**Supplementary Table 1**　Correlation between psoas muscle gauge and baseline variables

|  | Univariate analysis | | Multivariate analysis | |
| --- | --- | --- | --- | --- |
| Variables | r (95% confidence interval) | p-value | β (95% confidence interval) | p-value |
| Men | 0.409 (0.285 – 0.530) | <0.0001 | 0.361 (0.243 – 0.477) | <0.0001 |
| Age | -0.447 (-0.547 – -0.333) | <0.0001 | -0.325 (-0.473 – -0.176) | <0.0001 |
| Cardiovascular disease history | -0.147 (-0.280 – -0.014) | 0.030 | -0.029 (-0.135 – 0.077) | 0.59 |
| Modified creatinine index | 0.500 (0.393 – 0.594) | <0.0001 | 0.034 (-0.129 – 0.194) | 0.69 |
| Geriatric nutritional risk index | 0.444 (0.330 – 0.545) | <0.0001 | 0.216 (0.088 – 0.343) | 0.0010 |
| Log C-reactive protein | -0.251 (-0.371 – -0.121) | 0.0002 | -0.056 (-0.171 – 0.061) | 0.35 |

**Supplemental Table 2**Cox proportional hazards analysis of psoas muscle index (lower psoas muscle index vs. higher psoas muscle index) for all-cause mortality and new cardiovascular events in subgroups with gender and age

| Endpoints | Type of cutoff | **Subgroups** | No. event /total No. | Hazard ratio (95% confidence interval) | p-value |
| --- | --- | --- | --- | --- | --- |
| *All-cause mortality* |  |  |  |  |  |
|  | Asian^a^ |  |  |  |  |
|  |  | Gender |  |  |  |
|  |  | Male | 56/144 | 5.06 (2.29–11.20) | <0.0001 |
|  |  | Female | 29/73 | 2.31 (0.80–6.65) | 0.089 |
|  |  | Age |  |  |  |
|  |  | <65 years | 24/103 | 4.44 (1.98–9.93) | 0.0003 |
|  |  | ≥65 years | 61/114 | 2.41 (1.41–4.12) | 0.0013 |
|  | Median^b^ |  |  |  |  |
|  |  | Gender |  |  |  |
|  |  | Male | 56/144 | 4.02 (2.19–7.40) | <0.0001 |
|  |  | Female | 29/73 | 3.33 (1.48–7.50) | 0.0037 |
|  |  | Age |  |  |  |
|  |  | <65 years | 24/103 | 2.36 (1.06–5.30) | 0.037 |
|  |  | ≥65 years | 61/114 | 2.86 (1.49–5.51) | 0.0016 |
|  | ROC-derived^c^ |  |  |  |  |
|  |  | Gender |  |  |  |
|  |  | Male | 56/144 | 4.60 (2.69–7.90) | <0.0001 |
|  |  | Female | 29/73 | 3.86 (1.76–8.45) | 0.0007 |
|  |  | Age |  |  |  |
|  |  | <65 years | 24/103 | 4.44 (1.98–9.93) | 0.0003 |
|  |  | ≥65 years | 61/114 | 2.41 (1.41–4.12) | 0.0013 |
| New cardiovascular events |  |  |  |  |  |
|  | Asian^a^ |  |  |  |  |
|  |  | Gender |  |  |  |
|  |  | Male | 61/144 | 4.86 (2.39–9.89) | <0.0001 |
|  |  | Female | 34/73 | 1.19 (0.54–2.64) | 0.67 |
|  |  | Age |  |  |  |
|  |  | <65 years | 32/103 | 2.15 (1.03–4.62) | 0.041 |
|  |  | ≥65 years | 63/114 | 2.45 (1.11–5.44) | 0.027 |
|  | Median^b^ |  |  |  |  |
|  |  | Gender |  |  |  |
|  |  | Male | 61/144 | 3.63 (2.07–6.39) | <0.0001 |
|  |  | Female | 34/73 | 1.92 (0.96–3.86) | 0.067 |
|  |  | Age |  |  |  |
|  |  | <65 years | 32/103 | 2.45 (1.22–4.94) | 0.012 |
|  |  | ≥65 years | 63/114 | 1.94 (1.08–3.47) | 0.026 |
|  | ROC-derived^c^ |  |  |  |  |
|  |  | Gender |  |  |  |
|  |  | Male | 61/144 | 2.41 (1.44–4.02) | 0.0008 |
|  |  | Female | 34/73 | 2.78 (1.36–5.54) | 0.0047 |
|  |  | Age |  |  |  |
|  |  | <65 years | 32/103 | 3.31 (1.56–7.04) | 0.0019 |
|  |  | ≥65 years | 63/114 | 1.40 (0.85–2.30) | 0.19 |

^a^Cut-off values based on the psoas muscle index values of the healthy young Asian population: women, psoas muscle index of <4.91 cm^2^/m^2^; men, psoas muscle index of <6.75 cm^2^/m^2^

^b^Cut-off values based on the median values of psoas muscle index in this cohort: women, psoas muscle index of <4.11 cm^2^/m^2^; men, psoas muscle index of <5.86 cm^2^/m^2^

^c^Cut-off values based on the values obtained by ROC curve analysis for maximally predicting all-cause mortality: women, psoas muscle index of <3.39 cm^2^/m^2^; men, psoas muscle index of <4.98 cm^2^/m^2^

**Supplemental Table 3**Cox proportional hazards analysis of psoas muscle gauge (lower psoas muscle gauge vs. higher psoas muscle gauge) for all-cause mortality and new cardiovascular events in subgroups with gender and age

| Endpoints | Type of cutoff | **Subgroups** | No. event /total No. | Hazard ratio (95% confidence interval) | p-value |
| --- | --- | --- | --- | --- | --- |
| *All-cause mortality* |  |  |  |  |  |
|  | Asian^a^ |  |  |  |  |
|  |  | Gender |  |  |  |
|  |  | Male | 56/144 | 36.72 (5.07–265.96) | 0.0004 |
|  |  | Female | 29/73 | 4.83 (1.14–20.38) | 0.032 |
|  |  | Age |  |  |  |
|  |  | <65 years | 24/103 | 9.08 (2.13–38.66) | 0.0028 |
|  |  | ≥65 years | 61/114 | 14.16 (1.96–102.48) | 0.0087 |
|  | Median^b^ |  |  |  |  |
|  |  | Gender |  |  |  |
|  |  | Male | 56/144 | 9.64 (4.35–21.33) | <0.0001 |
|  |  | Female | 29/73 | 5.93 (2.35–14.98) | 0.0002 |
|  |  | Age |  |  |  |
|  |  | <65 years | 24/103 | 6.53 (2.67–15.93) | <0.0001 |
|  |  | ≥65 years | 61/114 | 5.43 (2.33–12.65) | <0.0001 |
|  | ROC-derived^c^ |  |  |  |  |
|  |  | Gender |  |  |  |
|  |  | Male | 56/144 | 4.61 (2.69–7.90) | <0.0001 |
|  |  | Female | 29/73 | 3.86 (1.76–8.45) | 0.0007 |
|  |  | Age |  |  |  |
|  |  | <65 years | 24/103 | 4.44 (1.98–9.93) | 0.0003 |
|  |  | ≥65 years | 61/114 | 2.41 (1.41–4.12) | 0.0013 |
| New cardiovascular events |  |  |  |  |  |
|  | Asian^a^ |  |  |  |  |
|  |  | Gender |  |  |  |
|  |  | Male | 61/144 | 7.17 (3.07–16.75) | <0.0001 |
|  |  | Female | 34/73 | 2.15 (0.83–5.59) | 0.12 |
|  |  | Age |  |  |  |
|  |  | <65 years | 32/103 | 3.05 (1.37–6.80) | 0.0064 |
|  |  | ≥65 years | 63/114 | 5.03 (1.56–16.26) | 0.0069 |
|  | Median^b^ |  |  |  |  |
|  |  | Gender |  |  |  |
|  |  | Male | 61/144 | 4.50 (2.53–8.03) | <0.0001 |
|  |  | Female | 34/73 | 2.67 (1.30–5.48) | 0.0076 |
|  |  | Age |  |  |  |
|  |  | <65 years | 32/103 | 3.53 (1.74–7.16) | 0.0005 |
|  |  | ≥65 years | 63/114 | 2.34 (1.27–4.34) | 0.0067 |
|  | ROC-derived^c^ |  |  |  |  |
|  |  | Gender |  |  |  |
|  |  | Male | 61/144 | 4.61 (2.62–8.10) | <0.0001 |
|  |  | Female | 34/73 | 3.39 (1.68–6.82) | 0.0006 |
|  |  | Age |  |  |  |
|  |  | <65 years | 32/103 | 3.97 (1.92–8.24) | 0.0002 |
|  |  | ≥65 years | 63/114 | 2.52 (1.45–4.38) | 0.0011 |

^a^Cut-off values based on the values of the healthy young Asian population: women, psoas muscle gauge of <231.1 arbitrary units (AU); men, psoas muscle gauge of <328.8 AU

^b^Cut-off values based on the median values of psoas muscle gauge in this cohort: women, psoas muscle gauge of <164.0 AU; men, psoas muscle gauge of <252.1 AU

^c^Cut-off values based on the values obtained by ROC curve analysis for maximally predicting all-cause mortality: women, psoas muscle gauge of <107.7 AU; men, psoas muscle gauge of <246.3 AU

**Supplementary Fig. 1** Kaplan–Meier survival curves for estimating cardiovascular event-free survival rate
The cardiovascular event-free survival rate for comparing the lower and higher psoas muscle index (PMI) groups based on the (a) sex-specific cut-off values of the healthy young Asian population^1^, (b) sex-specific median values^2^, and (c) sex-specific cut-off values obtained using the receiver operating characteristic (ROC) curve analysis^3^. The cardiovascular event-free survival rate for comparing lower and higher psoas muscle gauge (PMG) groups based on the (d) sex-specific cut-off values of the healthy young Asian population^4^, (e) sex-specific median values^5^, and (f) sex-specific cut-off values obtained using the ROC curve analysis^6^.

^1^Cut-off values based on the PMI values of the healthy young Asian population: women, PMI of <4.91 cm^2^/m^2^; men, PMI of <6.75 cm^2^/m^2^

^2^Cut-off values based on the median values of PMI in this cohort: women, PMI of <4.11 cm^2^/m^2^; men, PMI of <5.86 cm^2^/m^2^

^3^Cut-off values based on the values obtained using the ROC curve analysis for maximally predicting all-cause mortality: women, PMI of <3.39 cm^2^/m^2^; men, PMI of <4.98 cm^2^/m^2^

^4^Cut-off values based on the values of the healthy young Asian population: women, PMG of <231.1 arbitrary units (AU); men, PMG of <328.8 AU

^5^Cut-off values based on the median values of PMG in this cohort: women, PMG of <164.0 AU; men, PMG of <252.1 AU

^6^Cut-off values based on the values obtained using the ROC curve analysis for maximally predicting all-cause mortality: women, PMG of <107.7 AU; men, PMG of <246.3 AU
