# Supplementary material for: Psoas muscle gauge and adverse clinical outcomes in patients on hemodialysis
Source: J Nephrol. 2025 Jan 28;38(2):655–64. doi: 10.1007/s40620-024-02191-4 (PMC11961518; doi:10.1007/s40620-024-02191-4)
Supplement: Supplementary file 2 — Supplementary file2 (PPTX 87 KB) [file 40620_2024_2191_MOESM2_ESM.pptx]

## Slide 1
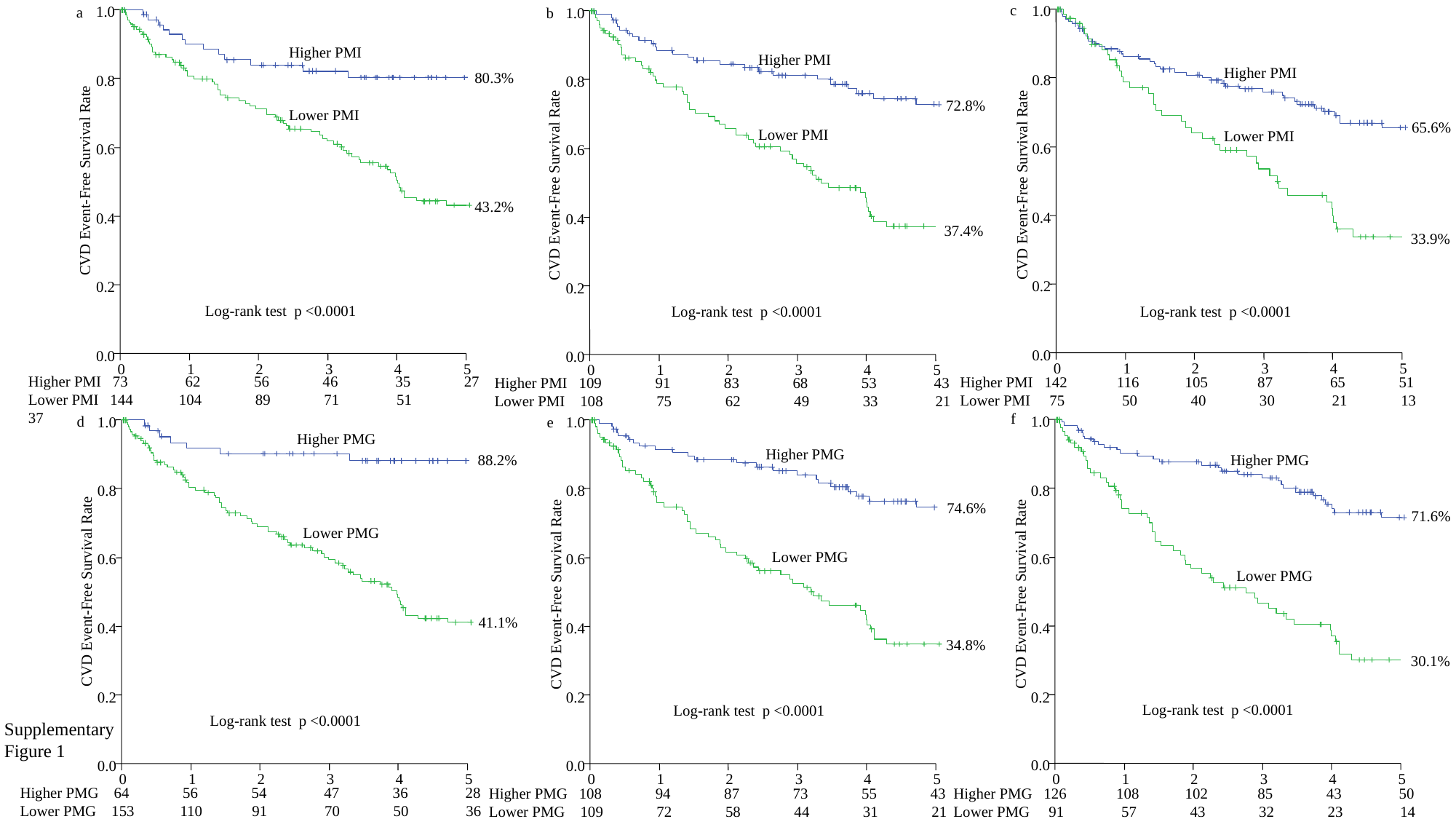

c
a
b
1.0
1.0
1.0
Higher PMI
Higher PMI
Higher PMI
80.3%
0.8
0.8
0.8
72.8%
Lower PMI
65.6%
Lower PMI
Lower PMI
0.6
0.6
0.6
CVD Event-Free Survival Rate
CVD Event-Free Survival Rate
CVD Event-Free Survival Rate
43.2%
0.4
0.4
0.4
37.4%
33.9%
0.2
0.2
0.2
Log-rank test p <0.0001
Log-rank test p <0.0001
Log-rank test p <0.0001
0.0
0.0
0.0
0
1
2
3
4
5
0
1
2
3
4
5
0
1
2
3
4
5
Higher PMI 73 62 56 46 35 27
Lower PMI 144 104 89 71 51 37
Higher PMI 142 116 105 87 65 51
Lower PMI 75 50 40 30 21 13
Higher PMI 109 91 83 68 53 43
Lower PMI 108 75 62 49 33 21
f
d
e
1.0
1.0
1.0
Higher PMG
Higher PMG
88.2%
Higher PMG
0.8
0.8
0.8
74.6%
71.6%
Lower PMG
Lower PMG
0.6
0.6
0.6
Lower PMG
CVD Event-Free Survival Rate
CVD Event-Free Survival Rate
CVD Event-Free Survival Rate
41.1%
0.4
0.4
0.4
34.8%
30.1%
0.2
0.2
0.2
Log-rank test p <0.0001
Log-rank test p <0.0001
Log-rank test p <0.0001
Supplementary
Figure 1
0.0
0.0
0.0
0
1
2
3
4
5
0
1
2
3
4
5
0
1
2
3
4
5
Higher PMG 64 56 54 47 36 28
Lower PMG 153 110 91 70 50 36
Higher PMG 126 108 102 85 43 50
Lower PMG 91 57 43 32 23 14
Higher PMG 108 94 87 73 55 43
Lower PMG 109 72 58 44 31 21
